# Metabolic features of innate lymphoid cells

**DOI:** 10.1084/jem.20221140

**Published:** 2022-10-27

**Authors:** Huiyang Yu, Nicolas Jacquelot, Gabrielle T. Belz

**Affiliations:** 1 The University of Queensland, Diamantina Institute, Brisbane, Queensland, Australia; 2 Princess Margaret Cancer Centre, University Health Network, Toronto, Ontario, Canada

## Abstract

Innate and adaptive immune cells are found in distinct tissue niches where they orchestrate immune responses. This requires intrinsic and temporal metabolic adaptability to coordinately activate the immune response cascade. Dysregulation of this program is a key feature of immunosuppression. Direct or indirect metabolic immune cell reprogramming may offer new approaches to modulate immune cells behavior for therapy to overcome dysregulation. In this review, we explored how metabolism regulates lymphocytes beyond the classical T cell subsets. We focus on the innate lymphoid cell (ILC) family, highlighting the distinct metabolic characteristics of these cells, the impact of environmental factors, and the receptors that could alter immune cell functions through manipulation of metabolic pathways to potentially prevent or treat various diseases.

## Teaser

Innate lymphoid cells are critical for tissue protection and immunity in animals, and their localization at the body’s surfaces position them to respond rapidly. Yu and colleagues review the metabolic pathways that underlie ILC phenotype and function and adaptation to dynamically changing conditions for homeostasis.

## Introduction

Evolutionary adaption has driven the development of an immune system that is classically divided into the innate and the adaptive arms. This division is based on the capacity of cells within these two different networks to respond to (i) specific antigens and (ii) to develop long-lived memory that might be protective during a second, or repeated, challenge with a pathogen or an inflammatory stimulus. In contrast to adaptive lymphocytes, innate immune cells such as innate lymphoid cells (ILCs) respond rapidly to environmental or pathogen threats through non-antigen–specific signals and fail to develop long-lived memory.

ILCs are enriched in mucosal tissues (e.g., lung and intestine) and within individual organs (e.g., liver and skin) but are poorly represented in circulation leading to them being known as “tissue-resident” cells. They are strategically positioned immediately adjacent to barrier tissues where they act as sentinels, or they are embedded in more distant organs, such as the liver, where they are exposed to circulating blood originating from the intestine. This enables ILCs to engage in surveillance, particularly of potential invading pathogens, and readily monitor homeostatic perturbations. Localization in these niches exposes ILCs to significant fluctuations in O_2_, nutrients, pH, blood supply, and other physiological factors (Caputa et al., 2019). Efficient activation of ILCs thus requires the capacity to rapidly adjust their biosynthetic and energy demands to local perturbations to meet their fluctuating metabolic requirements. Manipulation of immune cell metabolism, particularly T cells, offers great promise as an efficient targeting approach to tune immune cell function (Buck et al., 2017; Corrado and Pearce, 2022; Pålsson-McDermott and O’Neill, 2020). While nascent, the field of ILC metabolism has hitherto revealed key differences with T cells suggesting the potential to differentially harness T cells and ILCs in therapeutic settings.

## ILCs

The ILC family is composed of five subsets, namely, natural killer (NK) cells, ILC1s, ILC2s, ILC3s, and lymphoid tissue-inducer (LTi) cells, based on their distinct progenitors, developmental trajectories, transcription factor requirements, and cytokine expression (Vivier et al., 2018). ILC1s, ILC2s, and ILC3s mirror the cytokine and transcriptional profiles of helper cluster of differentiation (CD)4^+^ T cell subsets including T helper (Th)1, Th2, and Th17/22 cells, respectively. NK cells, in contrast, share similarities with CD8^+^ cytotoxic T cells due to their capacity to kill infected or malignant cells through the release of cytotoxic molecules. NK cells and ILC1s are characterized by the production of IFN-γ and dependence on the T-box transcription factor T-bet (encoded by *Tbx21*) for their development (Gordon et al., 2012). ILC1s are distinguished from NK cells by their expression of the α1-integrin subunit, CD49a, the IL-7 receptor, CD127, and the lack of expression of eomesodermin. ILC2s parallel Th2 cells through their secretion of the cytokines IL-5 and IL-13 (Neill et al., 2010; Vivier et al., 2018). They depend on GATA-binding protein 3 (GATA3) and retinoic acid–related orphan receptor (ROR)α for their development and maintenance (Ferreira et al., 2021; Furusawa et al., 2013; Hoyler et al., 2012; Wong et al., 2012). The expression of killer cell lectin-like receptor subfamily G member 1 (KLRG1) and suppressor of tumorigenicity 2 (ST2), a component of IL-33 receptor, divides ILC2s into two subsets, namely, natural (nILC2, KLRG1^−^ST2^+^) and inflammatory (iILC2, KLRG1^+^ST2^−^) ILC2s. At steady state, ILC2s in the intestine and specific adipose tissue compartments are mainly composed of iILC2 (Olguin-Martinez et al., 2021). These iILC2 are found in response to inflammation or IL-25 stimulation (Huang et al., 2015). ILC3s and LTi cells (members of the group 3 ILCs) depend on the transcription factor RORγt for their development and produce IL-17A and/or IL-22 (Satoh-Takayama et al., 2008; Takatori et al., 2009). ILC3s can be further divided by their expression of the natural cytotoxicity receptors (NCR) NKp46 (NCR1, in mouse and human) and NKp44 (NCR2, in human; Cupedo et al., 2009; Luci et al., 2009). ILC3 dysregulation and the associated increased production of IL-17A, IL-22, and IFN-γ can drive the development of inflammatory bowel diseases (IBD; Zeng et al., 2019). Distinct from ILC3s, LTi cells, which are crucial for the formation of secondary lymph nodes (Mebius et al., 1997), arise from a separate bone marrow progenitor (LTi progenitor [LTiP]; Vivier et al., 2018).

## Innate and adaptive immune cells: Shared and distinct metabolic programs

**Figure 1.**
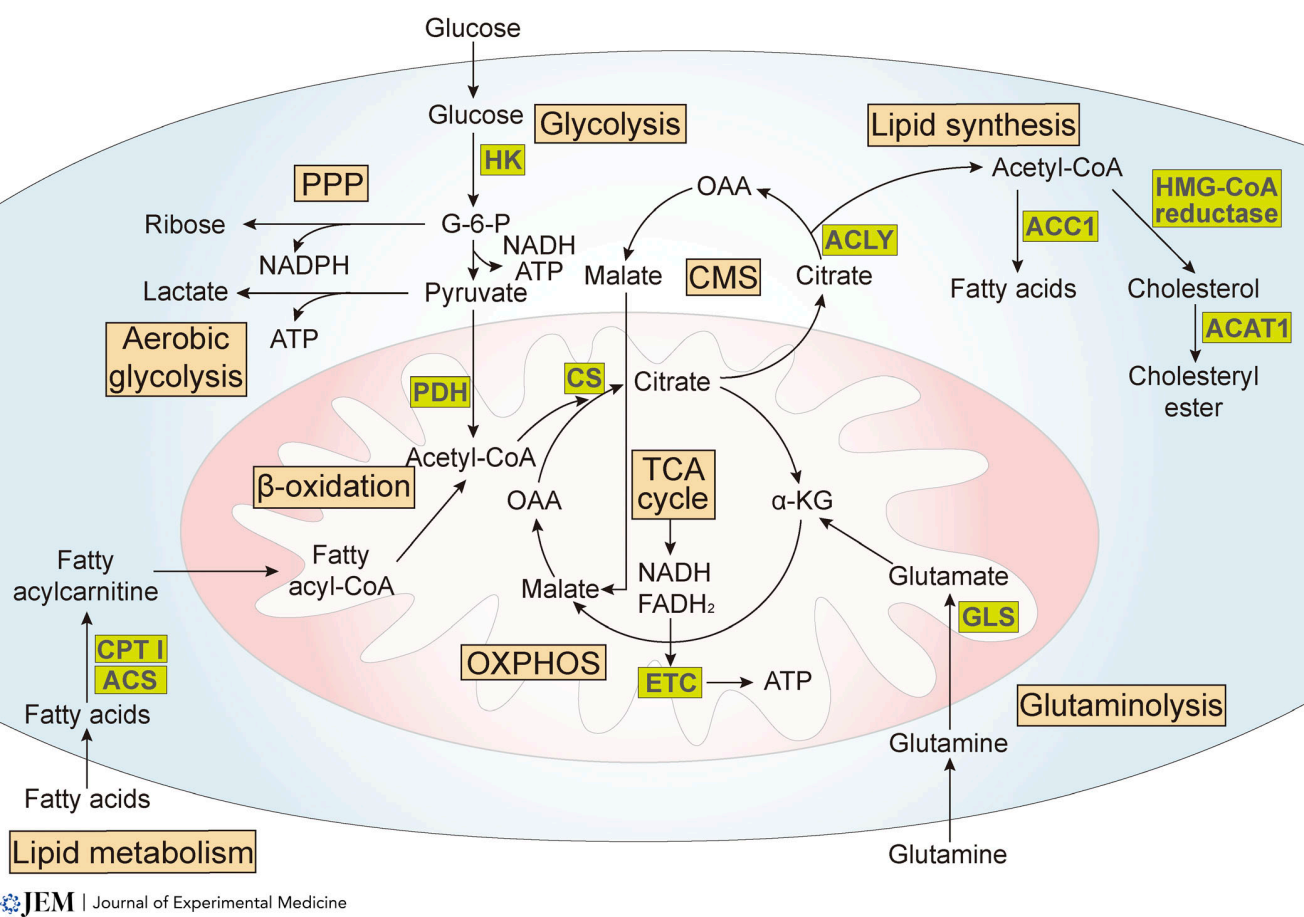
**Key metabolic pathways and factors in energy generation and biosynthesis.** Glucose metabolism starts with glycolysis. In the cytoplasm, glucose is converted into glucose-6-phosphate (G-6-P) catalyzed by HK and finally into pyruvate. Glycolysis also provides intermediates for the pentose-phosphate pathway (PPP), providing ribose as precursors for DNA and RNA synthesis, as well as nicotinamide adenine dinucleotide phosphate (NADPH) production. Pyruvate is transported into the mitochondria, where it is then converted into acetyl-CoA, catalyzed by pyruvate dehydrogenase (PDH). Acetyl-CoA enters the TCA cycle in mitochondria and forms citrate by reacting with oxaloacetate (OAA). Nicotinamide adenine dinucleotide (NADH) and flavin adenine dinucleotide (FADH_2_) are generated and then used to produce ATP via electron transport chain (ETC) in the process of OXPHOS. The need for ATP to generate energy is fundamental in both quiescent and activated immune cells (Pearce and Pearce, 2013). Increased aerobic glycolysis, also known as the Warburg effect, refers to the preferential use of glycolysis for ATP generation when sufficient oxygen is available, and is often associated with rapid cell proliferation (Warburg et al., 1927). Alteration of these pathways can influence the pro-inflammatory cytokines secreted by immune cells, especially macrophages, and trigger inflammatory responses that may be amplified by feedback loops and result in chronic disease (Lackey and Olefsky, 2016). Fatty acids can also fuel TCA cycle and OXPHOS to produce ATP. Fatty acids are converted into fatty acyl-CoA, catalyzed by fatty acyl-CoA synthetase (ACS) and CPT I (Iacobazzi et al., 2013), and then experience β-oxidation (also known as FAO), which converts fatty acyl-CoA into acetyl-CoA. Acetyl-CoA produced in the mitochondria must be transported within the cytosol for lipid synthesis or for other acetylation reactions through the CMS. Two key enzymes in the CMS are the ATP citrate lyase (ACLY), which cleaves citrate into acetyl-CoA and oxaloacetate in the cytosol, and the citrate synthase (CS), which catalyzes acetyl-CoA and oxaloacetate into citrate in the mitochondrion. ACLY links glucose and/or glutamine metabolism with FAS, and it has been shown to be upregulated or activated in response to rapidly proliferating cancer cells (Zaidi et al., 2012). FAS starts from the carboxylation of acetyl-CoA, catalyzed by ACC1 (Brownsey et al., 2006). 3-hydroxy-3-methyl glutaryl CoA (HMG-CoA) reductase is a key enzyme in cholesterol synthesis which converts acetyl-CoA into HMG-CoA (Roitelman et al., 1992). ACAT1 converts excess cellular cholesterol to cholesteryl esters. Statins, the inhibitors of HMG-CoA reductase, are widely used to treat atherosclerosis and have also been shown to reduce the severity of diseases in several preclinical models of auto-immunity and allo-immunity, principally altering helper T cell polarization and differentiation (Youssef et al., 2002; Zeiser et al., 2007). How such inhibitors that exert systemic influences affect metabolic changes of other immune cell subsets is currently less clear. Amino acids are the building blocks of immune cell life support, contributing to multiple intracellular metabolic pathways. Glutaminase (GLS) is a key enzyme in glutaminolysis, during which glutamine is first converted into glutamate, and then converted into α-ketoglutarate (α-KG), an intermediate metabolite of the TCA cycle. This pathway enables replenishment of substrates for the TCA cycle, a process known as anaplerosis, which is essential to sustain energy production (Yang et al., 2017).

Immune cells are instrumental in fortifying tissue barriers and fighting pathogens. Their capacity to achieve this is tightly linked to their cellular metabolism, determined by differential nutrient and oxygen availability across the body’s microenvironments (Ganeshan and Chawla, 2014; Fig. 1). Metabolic programming, and the potential to rewire cells, can be used to instruct the function and differentiation of different immune cell lineages including macrophages (Vats et al., 2006), dendritic cells (Everts et al., 2012), monocytes (Cheng et al., 2016), B cells (Caro-Maldonado et al., 2014), T cells (Ho et al., 2015), and ILCs (O'Sullivan and Sun, 2017). This ability to be manipulated offers great promise for strategic manipulation of immune cells in inflammation, infections, and cancer (Norata et al., 2015; Yang et al., 2016). The process of reprogramming itself involves alteration of the expression of nutrient transporters which not only mediate nutrient uptake, but also regulate energy production, biosynthesis, redox balance, and mitochondrial fitness.

**Figure 2.**
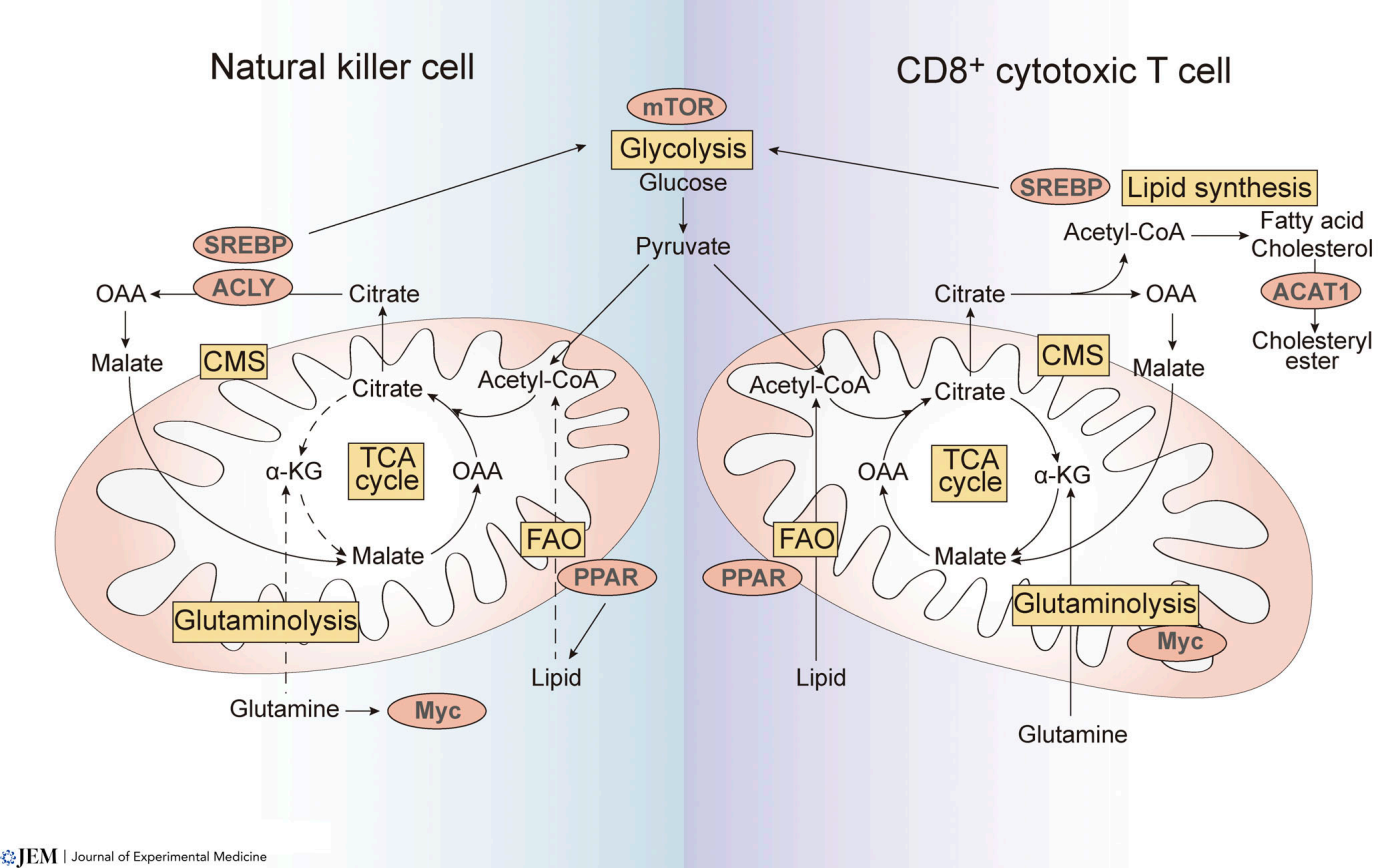
**Comparison of cellular metabolism and regulators in NK cells and CD8**^**+**^ **T cells.** Glycolysis and mTOR signaling are key to CD8^+^ cytotoxic T cell polarization and NK cell activation. The transcription factor Myc is regulated by mTOR signaling and plays an important role in both CD8^+^ T cells and NK cells. In CD8^+^ T cells, Myc controls glutaminolysis and links glutaminolysis with biosynthetic pathways, while in NK cells, the expression of Myc in murine NK cells is regulated by glutamine availability instead of glutaminolysis. Different from anaplerosis, in NK cells, glutamine does not supply TCA cycle and glutaminolysis does not sustain OXPHOS. In addition, NK cells and CD8^+^ T cells differ in their utilization of pyruvate. Pyruvate produced by glycolysis in NK cells directs metabolism into the CMS rather than the TCA cycle. SREBP expression in CD8^+^ cytotoxic T cells promotes lipid homeostasis during cellular expansion and enhances their glycolytic metabolism. In contrast, in NK cells, SREBP promotes CMS to control the level of glycolysis and OXPHOS. Thus, SREBP is now considered to be an important metabolic regulator of the glycolysis pathway in NK cells independent of its traditional role in lipid synthesis. ACAT1, a key cholesterol esterification enzyme, is essential for TCR engagement of CD8^+^ cytotoxic T cells, but whether ACAT1 plays a role in NK cell function remains unknown. PPARs, master regulators of lipid metabolism, play multiple roles in CD8^+^ T cell and NK cell metabolism. PPARs agonist could induce FAO in CD8^+^ T cells which enhances anti-tumor immunity and facilitates anti–PD-1 immunotherapy. In contrast, in obesity, PPAR-α/δ agonists leads to lipid accumulation in NK cells, impairing mTOR signaling, NK cell metabolism, and function. However, reduced NK cell function in B lymphoma caused by increased lipid metabolism could be partially restored by activating PPAR-γ. Furthermore, etomoxir (an inhibitor of FAO, blocking carnitine palmitoyltransferase 1, CPT I) does not affect ATP production in activated NK cells indicating that OXPHOS may not be predominantly fuelled by FAO (Keppel et al., 2015).

**Figure 3.**
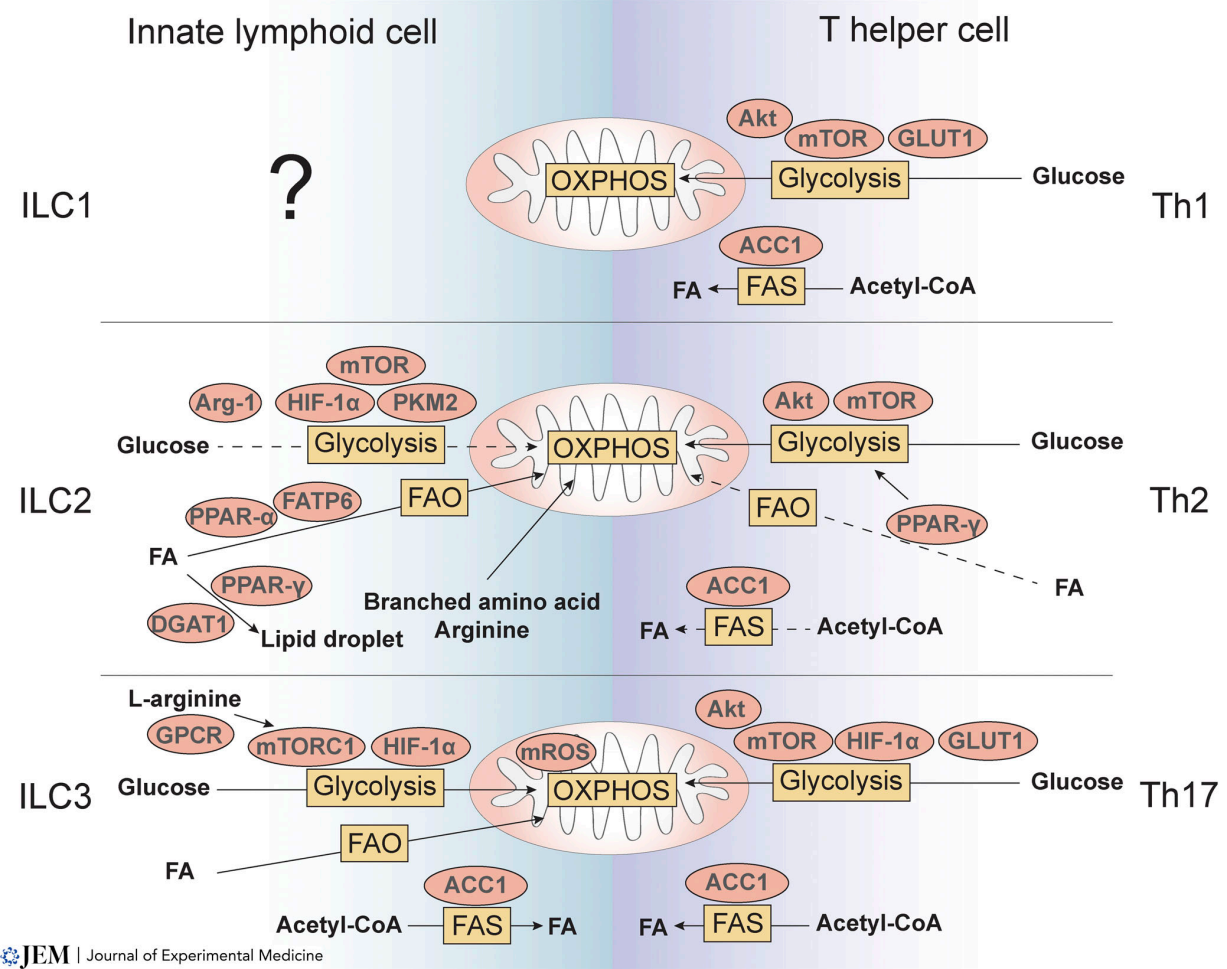
**Cellular metabolism and regulators in ILCs and Th cells.** Glucose metabolism drives the differentiation of CD4^+^ Th cells, which is regulated by the mTOR signaling pathway. Activated Th1, Th2, and Th17 cells rely on glycolysis and express high levels of GLUT1, a critical regulator of glycolysis in Th1 and Th17 cells, predominantly, although subset-dependent specificities also exist. While HIF-1α promotes Th17 cell polarization, PPAR-γ expression in Th2 cells enhances glycolysis and drives IL-9 production. In addition, ACC1-mediated de novo FAS is essential for the development of Th1 and Th17 cells in intestine as well as the pathogenic Th2 cells with high IL-33 expression and IL-5 production in lung and skin. ILC1 metabolism is largely unknown. However, NK cells can convert into ILC1-like cells mediated by TGF-β, which plays a role in the metabolic activity of cells. Thus, ILC1 metabolism could be linked to TGF-β–related metabolic activity changes, such as mTOR signaling and glucose and amino acid uptake. ILC2 preferentially metabolize fatty acids (FA) to fuel OXPHOS through FAO. Though glycolysis is not mainly utilized by ILC2, the level of glycolysis correlates with ILC2 function and is regulated by mTOR signaling, the glycolytic enzyme PKM2, and HIF-1α. Different from Th2 cells, the uptake of branched amino acids and arginine can also support OXPHOS in ILC2, which is required for ILC2 survival and IL-33–induced proliferation. Arg-1 is also a key metabolic checkpoint in ILC2, which promotes aerobic glycolysis and ILC2 proinflammatory function. Meanwhile, ILC2-derived IL-13 expression is mainly dependent on fatty acid uptake and FAO, through the upregulation of PPAR-α and fatty acid transport protein 6 (FATP6). ILC2 can also uptake external lipids and store them in lipid droplets, mediated by PPAR-γ and DGAT1. Like Th17 cells, mTORC1-HIF-1α axis regulates ILC3 proliferation and cytokine secretion. Similarly, ACC1 contributes to both ILC3 and Th17 effector function. In contrast, the level of mROS production in ILC3s is much higher than the level found in Th17 cells. This accumulation of mROS drives ILC3 activation, proliferation, and functions. In addition, distinct from Th17 cells, glucose and fatty acid metabolism are both upregulated in ILC3 after activation. Furthermore, G-protein-coupled receptor (GPCR) expression and L-arginine cytoplasmic levels in ILC3 participate in mTOR activation and influence colonic ILC3 proliferation and IL-22 production.

Although historically, ILCs have been considered as the innate counterparts of T cells (Vivier et al., 2016), recent evidence suggests that functionally, ILCs and T cells may utilize very different metabolic pathways to sustain their effector activity (Figs. 2 and 3; Table 1). The different effector functions of ILCs and T cells may contribute to their distinct metabolic strategies and reflect the adaptation of immune cells to distinct tissue niches. Like T cells, these metabolic pathways depend on the immune cell subset and their activation status.

**Table 1.** Metabolic pathways used by the innate and adaptive immune cells

Immune cell subset	Metabolic pathway(s)	Factor(s) regulated	Role in immune cell development and function	References
Cytotoxic CD8^+^ T cell	Glycolysis	mTORC1; Myc	Activation and polarization to gain killing ability	Araki et al., 2009; Wang et al., 2011
		SREBP	Heightened glycolytic metabolism and respiratory capacity during blastogenesis	Kidani et al., 2013
	FAO	PPAR	Increased FAO leading to enhanced anti-tumor immunity	Chowdhury et al., 2018
	FAS	ACAT1	Effector molecule production and TCR engagement	Yang et al., 2016
		SREBP; mTOR	Membrane synthesis, activation, and expansion	Kidani et al., 2013
	Glutaminolysis	Myc	Link with multiple biosynthetic pathways	Wang et al., 2011
NK cell	Glycolysis	mTOR; c-Myc	Effector molecule (especially IFN-γ) production; maintain killing ability and mitochondrial mass	Donnelly et al., 2014; Marçais et al., 2014; Düvel et al., 2010
		TGF-β; FBP1		Slattery and Gardiner, 2019; Zaiatz-Bittencourt et al., 2018
		SREBP	Effector molecule production and cytotoxicity (controlled through the CMS)	Assmann et al., 2017
	FAO	PPAR-α/δ	Effector molecule production; immunosurveillance against tumor growth; maintain mitochondrial mass and membrane potential	Michelet et al., 2018
		PPAR-γ		Kobayashi et al., 2020
Th1	Glycolysis	GLUT1; mTOR/Akt	Polarization and maintenance	Delgoffe et al., 2009; Macintyre et al., 2014
	FAS	ACC1	Expansion and infiltration	Mamareli et al., 2021
Th2	Glycolysis	mTOR/Akt	Polarization and maintenance	Delgoffe et al., 2009
	FAO	PPAR-γ	IL-9 expression	Micossé et al., 2019; von Meyenn et al., 2019
	FAS	ACC1	Development of IL-5–producing Th2 cells in lung and skin	Nakajima et al., 2021
Th17	Glycolysis	GLUT1; mTOR/Akt; HIF-1α	Polarization and proliferation	Delgoffe et al., 2009; Macintyre et al., 2014; Shi et al., 2011
	FAS	ACC1	Expansion and infiltration	Mamareli et al., 2021
ILC1	Unknown	TGF-β	ILC1-related plasticity	Fuchs et al., 2013
ILC2	FAO	PPAR-α; FATP6	Maintain IL-13 production in the context of helminth infection or malnutrition	Wilhelm et al., 2016
	Lipid synthesis	PPAR-γ; DGAT1	Lipid droplet formation, mitochondrial function, and proliferation	Karagiannis et al., 2020
	Glycolysis	mTOR	Control PPAR-γ and DGAT1 expression; proliferation and cytokine production	Karagiannis et al., 2020
		PKM2; HIF-1α	Induction of IL-33 receptor; maturation and function	Li et al., 2018; Surace et al., 2021a
		Arg-1	Glycolytic capacity, proliferation, and pro-inflammatory functions	Monticelli et al., 2016
	OXPHOS	Branched amino acid; arginine	Maintain cellular fitness and proliferation	Surace et al., 2021a
ILC3	Glycolysis	mTOR and ROS	Development, proliferation, and activation	Di Luccia et al., 2019
		HIF-1α	Cytokine production and switch to glycolysis	Fachi et al., 2021
	FAS	ACC1	RORγt expression; cytokine production and lipogenesis	Mamareli et al., 2021

PI3K, phosphoinositide 3-kinase; STAT6, signal transducer and activator of transcription 6; FBP1, gluconeogenic enzyme FBP1.

## Cytotoxic innate and adaptive lymphocyte subsets: CD8^+^ T cells and NK cells

CD8^+^ T cells and NK cells play a key role in tumor immunosurveillance, viral infection, and elimination of intracellular pathogens (Vivier et al., 2011). In the tumor microenvironment, however, the function of both these cells is progressively blunted in part due to their poor ability to infiltrate the densely packed tumor cells and the hypoxic environment (Clever et al., 2016; Eil et al., 2016; Geiger et al., 2016; Sukumar et al., 2017). CD8^+^ T cells can be metabolically reprogrammed to enhance oxidative phosphorylation (OXPHOS) by exogenous treatment with coenzyme A (CoA), by inducing the expression of glutamine transporters or supplementation with acetate to drive availability of acetyl-CoA in mitochondria to facilitate anti-tumor immunity (Song et al., 2018; St Paul et al., 2021). Similarly, metabolic reprogramming of NK cells to use glucose, lipids, and glutamine can also be induced, paving a pathway to improve NK cell killing functions and anti-tumor immunity (Poznanski et al., 2021). However, differences exist in the nutrients and metabolic pathways that are utilized by NK cells and CD8^+^ cytotoxic T cells to drive their behavior and function (Fig. 2).

The mammalian target of rapamycin (mTOR)–Myc proto-oncogene (Myc) and sterol regulatory element–binding protein (SREBP) pathways play a significant role in T cell and NK cell activation, expansion, and maturation (Saxton and Sabatini, 2017; Loftus et al., 2018; Wang et al., 2011). mTOR activity determines both the level of glycolysis and OXPHOS, influencing the generation of long-lasting CD8^+^ memory T cells (He et al., 2011). Similarly, activated NK cells depend on mTOR complex 1 (mTORC1) for increased glucose uptake and glycolysis levels, determining NK cell effector function (Donnelly et al., 2014). In T cells, Myc controls glutaminolysis and links glutaminolysis with biosynthetic pathways (Wang et al., 2011). In contrast, in NK cells, glutaminolysis and tricarboxylic acid cycle (TCA cycle) do not sustain OXPHOS. However, intracellular glutamine accumulation controls cMyc expression which affects NK cell growth and responses (Loftus et al., 2018). Lipid homeostasis in activated CD8^+^ cytotoxic T cells is maintained by SREBP which also enhances glycolytic metabolism and mitochondrial respiration (Kidani et al., 2013). In NK cells, pyruvate produced by glycolysis directs metabolism into the citrate-malate shuttle (CMS) rather than the TCA cycle (Assmann et al., 2017), a mechanism promoted by SREBP. This pathway controls the level of glycolysis and OXPHOS and is an essential metabolic regulator in NK cells (Assmann et al., 2017). The function of SREBP in NK cells is independent of its role described in CD8^+^ T cells (Kidani et al., 2013) or the role of regulating fatty acid and cholesterol synthesis in liver (Horton et al., 2002).

Cytokines play a pivotal role in tailoring the metabolic machinery of T cells and NK cells and differentially influence their metabolism and outcome (summarized in Table 2). While these features are extensively reviewed elsewhere (Cong, 2020; Kobayashi and Mattarollo, 2019; O’Brien and Finlay, 2019; Poznanski and Ashkar, 2019), it is clear that murine NK cells dramatically upregulate glucose uptake and glycolysis following activation with cytokines which favors upregulation of effector functions. Many of the changes observed relied on cMyc expression and mTORC1 signaling (Loftus et al., 2018). NK cell dysfunction could be rescued by prolonged treatment with high-dose IL-15 (Keppel et al., 2015), a cytokine required for both memory CD8^+^ T cell survival and NK cell development and maintenance (Berard et al., 2003; Cooper et al., 2002; Huntington et al., 2008). Interestingly, the dose of IL-15 directly determines the level of mTOR signaling and NK cell development in both humans and mice (Eckelhart et al., 2011; Felices et al., 2018). In addition to mTOR, other factors such as acetyl-CoA acetyltransferase 1 (ACAT1), a key cholesterol esterification enzyme important in receptor clustering (e.g., TCR; Yang et al., 2016) or peroxisome proliferator-activated receptors (PPARs, composed of PPARα, PPARγ, and PPARβ/δ) have been shown to play important roles in CD8^+^ T cell and/or NK cell metabolism (Christofides et al., 2021). In cancer, PPAR agonist administration induces fatty acid oxidation (FAO) in CD8^+^ T cells, enhancing anti-tumor immunity, and facilitates anti-programmed cell death protein 1 (PD-1) immunotherapy (Chowdhury et al., 2018). In NK cells, PPAR inhibition restores NK cell cytotoxicity (Michelet et al., 2018) while activation of PPAR-γ could overcome diminished cytokine production and killing ability (Kobayashi et al., 2020). Although the relationship between increased FAO and impaired NK cell function and signaling by different PPAR subtypes on NK cells requires further exploration, it is evident that mTOR, Myc, SREBP, PPARs, and the cytokine IL-15 differentially influence CD8^+^ T cell and NK cell metabolic programs and effector function.

**Table 2.** NK cell activation and metabolic modulation in response to different stimuli

Stimuli	Dosage	NK cell response	Source of NK cells	Reference
High-dose or low-dose IL-12 and IL-18	IL-12 (1 ng/ml), IL-18 (1 ng/ml); or IL-12 (10 ng/ml), IL-18 (50 ng/ml)	Activation and IFN-γ production independent of glycolysis or OXPHOS	Splenocytes (murine)	Keppel et al., 2015
Anti-NK1.1 or anti-Ly49D	20 μg/ml	Activation and IFN-γ production dependent on glycolysis and OXPHOS	Splenocytes (murine)	Keppel et al., 2015
High-dose IL-15 and anti-NK1.1 or anti-Ly49D	IL-15 (100 ng/ml), anti-NK1.1, or anti-Ly49D (20 μg/ml)	Activation and IFN-γ production independent of glycolysis or OXPHOS	Splenocytes (murine)	Keppel et al., 2015
High-dose IL-15	IL-15 (100 ng/ml) or IL-15/IL-15Rα complex (50 or 100 ng/ml)	Activation, IFN-γ production, and increased metabolic profile (glycolysis and OXPHOS) dependent on mTOR signaling	Splenocytes (murine)	Marçais et al., 2014; Nandagopal et al., 2014
Low-dose IL-15	IL-15 (10 ng/ml) or IL-15/IL-15Rα complex (10 ng/ml)	Cell survival and viability dependent on STAT5 phosphorylation	Splenocytes (murine)	Marçais et al., 2014; Nandagopal et al., 2014
IL-2 and IL-12	IL-2 (20 ng/ml), IL-12 (10 ng/ml)	Activation and IFN-γ production; increased metabolic profile (glycolysis and OXPHOS) independent of glutamine-fueled TCA cycle; increased cMyc expression dependent on glutamine availability and SLC7A5 activity	Splenocytes (murine)	Loftus et al., 2018
IL-12, IL-15, and IL-18 (CIML NK cells)	IL-12 (10 ng/ml), IL-15 (10 ng/ml), IL-18 (50 ng/ml)	More IFN-γ production after re-stimulated by IL-12 (10 ng/ml) and IL-15 (100 ng/ml) or anti-Ly49H or anti-NK1.1 (5 μg/ml)	Splenocytes (murine)	Cooper et al., 2009
	IL-12 (10 ng/ml), IL-15 (100 ng/ml), IL-18 (50 ng/ml)	More IFN-γ production after re-stimulated by IL-12/15/18 (10, 100, 50 ng/ml, respectively) or K562 cells; shift towards glycolysis upon activation with short-term increased OXPHOS; increased expression of nutrient transporters (CD98 and GLUT1)	PBMC (human)	Terrén et al., 2021
IL-12 and IL-15	IL-12 (30 ng/ml), IL-15 (100 ng/ml)	Activation and IFN-γ production dependent on elevated OXPHOS; upregulated glycolysis and OXPHOS insensitive to rapamycin	PBMC (human)	Keating et al., 2016
IL-2	500 U/ml	Activation and IFN-γ production dependent on elevated OXPHOS; upregulated glycolysis and OXPHOS dependent on mTORC1	PBMC (human)	Keating et al., 2016
IL-2 and anti-NKG2D	IL-2 (200 U/ml), anti-NKG2D (5 μg/ml)	Activation and IFN-γ production; upregulated expression of SLC1A5 and CD98 mediated by mTORC1 (a prerequisite of following NKG2D-mediated activation)	PBMC (human)	Jensen et al., 2017

CIML, cytokine-induced memory-like; SLC7A5, solute carrier family 7 member 5; SLC1A5, solute carrier family 1 member 5.

## Helper adaptive and innate lymphocyte subsets: CD4^+^ T cells and type 1, 2, and 3 ILCs

How metabolism supports and controls CD4^+^ T cell function has been explored in significant detail and has revealed that even within a single immune cell lineage, diversity of metabolic programs is required to support their diverse functions (Buck et al., 2017; Chapman et al., 2020; Geltink et al., 2018; MacIver et al., 2013; Pearce et al., 2013). Naive T cells mainly take up glucose and use OXPHOS to generate energy through ATP production, while activated T cells, which express high levels of glucose transporter 1 (GLUT1; Michalek et al., 2011), utilize a combination of glycolysis and OXPHOS to sustain their effector functions (MacIver et al., 2013). Increased glycolysis, through the aerobic glycolytic pathway, is regulated by mTOR signaling (Delgoffe et al., 2009; Ganeshan and Chawla, 2014; Zheng et al., 2007) and drives effector functions (e.g., cytokine production) rather than proliferation, accounting for CD4^+^ T cell utilization of this less efficient metabolic pathway for their activation (Chang et al., 2013). In addition to glycolysis and OXPHOS, Th17 cells also rely on fatty acid synthesis (FAS), whereas regulatory T cells (Tregs) utilize FAO for their functions. This contrasts with memory T cells which rely on FAO to support OXPHOS and their energy demands (Geltink et al., 2018).

### Type 1 ILCs

NK cells and ILC1 are typically considered to be distinct lineages. However, in tumors, NK cells can transdifferentiate into ILC1-like cells (CD49a^+^CD49b^−^), a process mediated by TGF-β, and which is accompanied by a downregulation of eomesodermin (Cortez et al., 2017; Gao et al., 2017). In this setting, TGF-β limits IL-15–induced NK cell activation by inhibiting mTOR and their metabolic activity, including glucose uptake, expression of amino acid transporters, and the level of glycolysis and OXPHOS (Viel et al., 2016). This suggests that “plasticity” might be highly manipulatable and mediated by metabolic reprogramming to shape the phenotype and function of NK cells and ILC1 cells to promote an anti-tumor program. While we have begun to understand aspects of NK cell metabolism, much more needs to be understood about the metabolic regulation of ILC1 and transdifferentiated cells.

### Type 2 ILCs

While Th2 cell metabolism is regulated by mTOR and Akt and engages glycolysis (Delgoffe et al., 2009; Stark et al., 2019), ILC2s at barrier sites take up long-chain fatty acids and preferentially metabolize fatty acids to fuel OXPHOS through FAO, promoting IL-13 expression during helminth infections (Wilhelm et al., 2016). However, ILC2 can also increase their glycolysis and oxygen consumption and use branched chain amino acids and arginine to support OXPHOS and to maintain their cellular fitness (Surace et al., 2021a). When activated, both T cells and ILC2 upregulate PD-1 expression, altering their metabolic program and impairing their effector function (Helou et al., 2020; Jacquelot et al., 2021; Moral et al., 2020; Patsoukis et al., 2015). PD-1 deficiency in ILC2 reprograms their metabolism toward glycolysis and glutaminolysis, enhancing their proliferation and cytokine production (Helou et al., 2020; Jacquelot et al., 2021). Furthermore, PPAR-γ influences both Th2 cells and ILC2 metabolic programs. In T cells, PPAR-γ regulates IL-9 production (Micossé et al., 2019) and promotes glycolysis (von Meyenn et al., 2019) while in ILC2s, PPAR-γ supports IL-33–mediated ILC2 pro-tumoral functions by increasing IL-13 secretion (Ercolano et al., 2021). Moreover, PPAR-γ and diacylglycerol acyltransferase 1 (DGAT1) promotes the accumulation and storage of lipids in ILC2 within droplets to promote ILC2 proliferation (Karagiannis et al., 2020). In addition, Arginase-1 (Arg-1) expression in ILC2 promotes aerobic glycolysis and controls ILC2 proliferation and cytokine production (Monticelli et al., 2016).

### Type 3 ILCs

Similar to activated Th17 cells (Delgoffe et al., 2009; Gerriets et al., 2015; Shi et al., 2011), the mTORC1-hypoxia-inducible factor (HIF)–1α axis and acetyl-CoA carboxylase 1 (ACC1) regulates ILC3 proliferation and cytokine secretion (Di Luccia et al., 2019; Mamareli et al., 2021). Like Th17 cells, mTORC1 expression in ILC3 activates HIF-1α and promotes glycolysis, a prerequisite to IL-17A and IL-22 production (Di Luccia et al., 2019). In colonic ILC3s, mTORC1 activation is regulated by G-protein-coupled receptor expression and L-arginine cytoplasmic levels, which influence both cell proliferation and IL-22 production (Hou et al., 2022). Although similarities exist between Th17 and ILC3, the comparison of their metabolic pathways has revealed a unique ILC3-dependent metabolic program that relies on mitochondrial ROS (mROS) production for their activation (Di Luccia et al., 2019). Indeed, the level of mROS in Th17 is typically low and mROS expression generally drives T cell differentiation toward the Treg subset (Gerriets et al., 2015). However, in ILC3, mROS is essential for their activation and, together with mTORC1, they sustain the expression of RORγt which supports ILC3 development, proliferation, and function (Di Luccia et al., 2019). When ILC3 are activated by cytokines (IL-1β, IL-23) or following pathogen infection (*Citrobacter rodentium*), the glucose and fatty acid uptakes are both increased (Di Luccia et al., 2019). In response to dextran sulfate sodium–induced colitis, LTi cells not only increased their glycolysis and FAS but also exhibited enhanced PD-1 expression. While PD-1 deficiency alters ILC3 metabolism toward an increase of FAO and reduced effector functions compared to their WT counterparts, the blockade of FAO rescues IL-22 production in PD-1–deficient ILC3 (Wu et al., 2022). Supporting these findings, transcriptional and epigenetic mapping of intestinal ILCs has shown that ILC3s were enriched in pathways related to glycolysis (Gury-BenAri et al., 2016).

Collectively, it is evident that ILCs share a number of similarities in metabolic programming with their adaptive counterparts. However, it is emerging that each ILC subset utilizes a unique set of regulators and signaling pathways to establish their metabolic profile directly influencing their effector function.

## Metabolic programming: Exhaustion, trained immunity, and plasticity

### CD8^+^ T cell and NK cell exhaustion

Immune cells, under certain circumstances such as chronic infections or during cancer (Wherry and Kurachi, 2015), exhibit features of lymphocyte exhaustion. In CD8^+^ T cells, this is associated with reduced expression of genes related to TCA cycle, energy metabolism, and solute and ion channels (Wherry et al., 2007). Increased cholesterol in CD8^+^ T cells has been shown to result in an exhausted phenotype in the tumor microenvironment (Ma et al., 2019). Such a phenotype could be reversed using an engineered IL-2 partial agonist, H9T which could alter STAT5 signaling and reduce the level of glycolysis in CD8^+^ T cells (Mo et al., 2021). Limitations on the availability in amino acids sustain NK cell exhaustion. Supplementation of cultures with L-arginine restores the IFN-γ production of exhausted NK cells when co-cultured with CD33^+^ human peripheral blood mononuclear cells (PBMCs) from hepatitis C virus–infected patients (Goh et al., 2016), prompting the use of amino acid supplementation for therapeutic intervention.

### Trained memory in innate lymphocytes

Trained immunity leads to the change in the metabolism, epigenetic, and transcription factor expression of innate lymphocytes and thus confers the capacity for faster and more robust responses to re-encounter with an antigen, similar to hallmark features of memory cells in the adaptive immune system (Dominguez-Andres et al., 2019; Netea et al., 2020). This cellular plasticity enables rapid metabolic adaption of innate lymphocytes at the front line of defense to significantly enhance immune protection and the potential to be harnessed for therapeutic approaches (Imran et al., 2020; Sohrabi et al., 2020). Recent findings have shown that IL-33 intranasal administration, or *C. rodentium* infection, induced long-term lung ILC2 and intestinal ILC3 cellular reprogramming, respectively, allowing them to respond more potently to a second challenge (Martinez-Gonzalez et al., 2016; Serafini et al., 2022). NK cell–adaptive-like responses have been also observed during viral infections, hapten exposure, and upon cytokine stimulation (Mikelez-Alonso et al., 2021). The metabolic profile of trained innate lymphocytes remains largely unknown. However, recent evidence suggests that trained ILC3s have increased OXPHOS compared to their naive counterparts (Serafini et al., 2022). In addition, cytomegalovirus-induced memory NK cell formation rely on OXPHOS (Surace et al., 2021b), while detailed analyses of cytokine-induced memory NK cells have revealed increased glycolysis and elevated expression of CD98 and GLUT1 (Terrén et al., 2021) attesting of metabolic changes during NK cell memory induction.

### CD4^+^ T cell and ILC plasticity

CD4^+^ T cell subsets are known to readily convert into other phenotypes, a phenomenon known as plasticity, and observed commonly in Th17 cells and Tregs (Zhou et al., 2009). IL-1β and IL-6 stimulation converts Tregs into Th17 cells, a process that could be blocked by TGF-β (Beriou et al., 2009). When activated by IL-12 or IL-4, Th17 cells can produce IFN-γ or IL-4, which are the functional features of Th1 and Th2 cells, respectively (Zhou et al., 2009).

ILC plasticity was originally discovered in RORγt^+^ILC3s which could differentiate into ILC1-like cells driven by IL-12 and IL-15 stimulation. These cells, known as ex-RORγt^+^ ILC3s, were able to release IFN-γ and induce inflammation, similar to group 1 ILCs (Vonarbourg et al., 2010). More recently, differentiation from ILC3 to CD127^+^ ILC1s has been observed, and showed an increase in the number of ILC1s in inflamed intestinal tissues at the cost of ILC3s (Bernink et al., 2015; Goc et al., 2021). Furthermore, ILC2s have been shown to convert into an IFN-γ–producing ILC1 phenotype cells in response to IL-12 stimulation (Ohne et al., 2016), a feature first described in chronic obstructive pulmonary disease. This phenotype could be reversed by supplementing cells in vitro with IL-4 (Bal et al., 2016). Lastly, the transition from ILC2 to ILC3-like cells has been observed in skin ILCs during psoriatic inflammation (Bielecki et al., 2021). As plasticity is usually induced by cytokines during inflammation, metabolic changes will inevitably accompany phenotypic, functional, and transcriptional changes in these cells.

## Environmental modulators and potential targets

### Microbiota

*Lactobacillus*, a gram-positive bacteria and a major component of surface biofilms, has been shown to degrade dietary tryptophan to indole derivatives which activate the transcription factor aryl hydrocarbon receptor (AhR) driving AhR-dependent *Il22* transcription in intestinal ILC3s (Zelante et al., 2013). Through regulating tryptophan metabolism and IL-22 expression, this microbiota–AhR axis tunes mucosal reactivity (Zelante et al., 2013). Analysis of transcriptional profiles of ILC subsets isolated from germ-free or specific pathogen–free mice that were exposed to antibiotics or left untreated has revealed an intricate relationship between microbiota and ILC function. In specific pathogen–free condition, ILC3 expressed high level of *Hk2* (encoding hexokinase [HK] 2), a key enzyme in the first step of glycolysis. *Hk2* expression was upregulated in ILC1 and ILC2 following antibiotic exposure (Gury-BenAri et al., 2016), suggesting that ILC metabolism could be regulated by microbiota. In addition, *Il17a* was lost in almost all ILC subsets following microbiota depletion, emphasizing the dependency of ILCs on commensal microbes for the induction of effector functions (Gury-BenAri et al., 2016). Thus, ILC functions are influenced by the composition of the intestinal microbiota and changes occurring in microbiota-derived metabolites that drive metabolic reprogramming of these cells.

### Oxygen availability

In the hypoxic environment, the transcription factor HIF is induced. When total oxygen is limiting, HIF-1 is able to trigger an increase of intracellular oxygen tension necessary to maintain the cell survival (Papandreou et al., 2006). This is achieved by upregulation of glycolysis through induction of GLUT1 and glycolytic enzymes such as HK1 and pyruvate kinase M (PKM; Semenza, 2003), and suppression of mitochondrial OXPHOS by downregulation of the expression of pyruvate dehydrogenase kinase 1 (PDK1; Papandreou et al., 2006).

In IL-33–activated ILC2s, the glycolytic enzyme PKM2 controls ILC2 maturation and development in peripheral non-lymphoid tissues by modulating ST2 expression in an HIF-1α–dependent manner (Surace et al., 2021a). Von Hippel-Lindau deficiency in ILCs resulted in accumulation of HIF-1α and decreased ST2 expression in lung ILC2s associated with reduced glycolysis and effector functions (Li et al., 2018). More recently, HIF-1α has been shown to control the plasticity of immature ILC2s differentiating into the ILC1-like IFN-γ–producing subset (Corral et al., 2021 *Preprint*). This plasticity was observed during *Mycobacterium tuberculosis* lung infection, suggesting that the glycolytic metabolic program driven by HIF-1α orchestrated adaption to the changes in ILC2 local environment (Corral et al., 2021 *Preprint*). In intestinal ILC3, HIF-1α expression stimulates their cytokine production independently of the glycolysis, mitochondrial respiration, or mTOR signaling (Fachi et al., 2021). In addition, HIF-1α promotes *Tbx21* expression in NKp46^+^ cells, driving RORγt^+^ILC3s differentiation into ILC1-like cells (Krzywinska et al., 2022).

HIF-1α has also been shown to play a broader role in shaping immune function. Monocytes stimulated by β-glucan exhibited “trained immunity,” with higher glucose consumption and lactate production, while blockade of the Akt-mTOR-HIF-1α pathway, or deficiencies in HIF-1α, blocked the induction of trained immunity in these cells (Cheng et al., 2014). Similarly, Tso et al. (2018) showed that the virulence of the opportunistic fungal pathogen *Candida albicans* in the mouse gut could be selected to favor the enhanced immune protection and enhanced ability to induce trained immunity, a process also dependent on the gut microbiota (Tso et al., 2018). Collectively, these findings highlight that hypoxia-mediated HIF-1α expression in innate immune cells drives subset-dependent metabolic reprogramming, necessary for the adaption of these cells to limited oxygen availability for their maintenance and activity.

An increased hypoxic environment in solid tumors alters the capacity of immune cells to restrict cancer cell growth (DePeaux and Delgoffe, 2021). While hypoxia induces mitochondrial defects through Myc regulation which drives T cell exhaustion (Liu et al., 2020), in contrast, HIF-1α expression enhances intratumor CD8^+^ T cell accumulation and effector functions (Doedens et al., 2013; Liikanen et al., 2021). In NK cells, hypoxia diminishes expression of activating receptors such as NKp46 and NK group 2D (NKG2D), impairing NK cell function (Balsamo et al., 2013) although deletion of HIF-1α correlated with increased IFN-γ and NK cell anti-tumor activity (Ni et al., 2020). An increase understanding of how HIF-1α and other metabolic regulators can modulate immune cells and change fate outcomes in diseases warrants further investigations.

## Future directions

Emerging findings have demonstrated that metabolic control in immune cells, particularly innate lymphocytes, may provide a largely untapped pathway for immunomodulation and immunotherapy. Metabolic checkpoints have been proposed for their potential use in anti-tumor or anti-inflammatory strategies (Guo et al., 2019; Pålsson-McDermott and O'Neill, 2020). These approaches have largely focused on molecules targeting specific metabolic pathways including glycolysis (targeting PDK, GLUT1, lactate dehydrogenase), OXPHOS (targeting Complex I in electron transport chain), and FAO (targeting carnitine palmitoyltransferase I [CPT I]; O'Neill et al., 2016; Zhang et al., 2018). However, an alternate approach is to manipulate receptors such as PD-1 which promote tumor-induced immunoregulation not only through their receptor–ligand interactions but also through the alteration of the metabolic framework for cells (Patsoukis et al., 2015). Given that ILCs and T cells diverge in many aspects as they differentially use glucose, fatty acids, and amino acids to undergo proliferation and effector activity, this provides a possibility to distinctively target different immune cells across tissues or within a tissue. This is particularly important as ILCs form a complex network of tissue-resident sentinel cells optimally positioned to coordinate integration of environmental cues with immune cell activation and effector responses. Integration of these complex signals establishes the set point for induction of innate cell differentiation and effector function and subsequently the type of response from adaptive cells. Tissue-specific tropisms that occur during infection are also a key factor in defining the metabolic programming of tissue-resident cells. These have evolved in parallel with infectious organisms to prevent organ dysfunction and failure and to promote a return to homeostasis.

Metabolic constraints in tissues where nutrients, oxygen, and cellular waste form gradients, influence the metabolism of tissue-resident and infiltrating immune cells. In the tumor microenvironment, low nutrient level, hypoxia, blockade of metabolic pathways, and suppressive metabolites contribute to impairing immune cell infiltration and effector function (DePeaux and Delgoffe, 2021). In mucosal and intestinal tissues, ILCs directly encounter dietary and microbial metabolites, which, together, establish them as therapeutically manipulatable targets on the front line. However, many unresolved questions currently exist in understanding ILC immunometabolism and how it could be harnessed to promote tissue repair or quench disease development.

### Microbial influences

The microbiome has been shown to alter the metabolism of ILC, potentially impacting tumor immunotherapy efficacy (Panda and Colonna, 2019). How the ILC–microbiome bidirectional signaling shapes barrier surface homeostasis and might expose the barrier to susceptibility to infection or inflammatory stimuli remains unclear. Conversely, direct targeting of ILCs may be an avenue to fortify the mucosal barrier or to alter the course of diseases such as IBD or asthma.

### Cellular adaptability/plasticity

ILC plasticity is often observed under inflammatory conditions. The link between plasticity and metabolism remains unclear. Future studies to unravel the mechanisms of ILC plasticity and whether induction of NK cells→ILC1, ILC2→ILC1, ILC2→ILC3, and ILC3→ILC1 re-programs metabolic wiring to temporally influence mucosal surface outcomes positively or negatively are needed. Future findings may define a new model of physiological regulation of homeostasis.

### Contextual diversity of ILC2 responses

ILC2 exhibit diverse outcomes in a tissue-specific manner accompanied by metabolic changes during inflammation, responses to parasite and pathogens and regulation of obesity and metabolic diseases such as diabetes. It will be important to understand how ILC2 are finely tuned to different tissues to respond rapidly to biosynthetic precursors and implement effector and divergent metabolic programs. For example, it has been shown that clinicopathogenesis of ILC2-driven airway inflammation can be altered using a ketogenic diet (Karagiannis et al., 2020). This suggests that diet could be used as a holistic approach to modulate ILC function.

### Complementarity of ILC1

Little is known about the metabolism of ILC1, particularly how those found in mucosal sites differ from the unique populations restricted to non-mucosal sites such as the liver. How these cells integrate signals to modulate their metabolism and mediate effector functions during tumor development, or infection, remains unknown. Importantly, in liver, where NK cells and ILC1 are found at similar frequencies, understanding of whether they share metabolic pathways or that these are distinct may open up new avenues for specific cellular reprogramming to improve disease control (Ducimetiere et al., 2021).

### Reprogramming disease outcomes

Metabolic reprogramming of immune cells is an attractive pathway to improve the efficacy of adoptively transferred CAR T cells (Pellegrino et al., 2020; Zhang et al., 2021). While currently applied to T cells, this approach could be applied to innate immune cell subsets such as ILCs that are engaged in an immune response early. Harnessing different ILC functions through metabolic pathways, alone, or in combination with immune checkpoint inhibitors could further improve anti-tumor capability. For example, reversing TGF-β–driven transdifferentiation of NK cells into ILC1-like cells, which are less proficient in tumor immunosurveillance, may provide a pathway to overcome tumor immunoevasion (Gao et al., 2017). Thus, harnessing a key pathway such as TGF-β may positively regulate NK cell metabolism, reinstating NK cell killing capacities and immune protection and immunotherapy options (Slattery and Gardiner, 2019).

Our increased understanding of the complementarity of adaptive and innate immune cell metabolism opens new avenues to therapeutically modulate immune cell function and influence disease outcomes. While increased evidence suggest that ILCs bear similar potential to adaptive cells such as T cells, we currently lack the in-depth knowledge necessary to harness ILC-dependent metabolic pathways in a subset-, tissue-, and disease-specific manner. Thus, future studies are urgently required to decipher the metabolic wiring of ILCs in health and diseases to take full advantage of their therapeutic potential in our immunotherapeutic armory.
